# A Stepwise Method to Derive Functional Pancreatic Islet‐like Clusters from Human Pluripotent Stem Cells

**DOI:** 10.1002/cpz1.70467

**Published:** 2026-09-25

**Authors:** Manuj Bandral, Jimena Alvarez‐Castanon, David S. Lorberbaum

**Affiliations:** ^1^ Department of Pharmacology University of Michigan Medical School Ann Arbor Michigan USA

**Keywords:** β‐cells, differentiation, islet, stem cell, Type 1 diabetes

## Abstract

The most common treatment for people with people with type 1 diabetes (T1D) requires injections of exogenous insulin to regulate blood glucose homeostasis. A longer‐term therapeutic solution for T1D has emerged whereby patients may regain insulin independence through transplantation of donated human pancreatic islets, which contain insulin‐secreting beta (β)‐cells. Donated islets, however, are in short supply. Much effort has been dedicated to using human pluripotent stem cell (hPSC)‐derived islets (SC‐islets) as an unlimited source of insulin‐secreting β‐cells. Here, we describe a robust and reproducible 40‐day, seven‐stage stepwise process for generating hPSC‐derived SC‐islets. Our differentiation protocol aims to mimic in vivo pancreas development using stage‐specific signals to promote the generation of SC‐islets. The initial maintenance and the first four stages are performed in two‐dimensional monolayer culture; the subsequent three stages require three‐dimensional culture in microwells to induce the formation of islet‐like clusters. The SC‐islets generated at the end of the procedure respond to glucose challenges, reliably express appropriate endocrine markers, contain intracellular insulin, and display an islet‐like architecture. Most SC‐islets also partially express maturity markers that are often challenging to activate in vitro. These protocols thus provide a scalable platform for generating SC‐islets for disease modeling and cell replacement studies. © 2026 The Author(s). *Current Protocols* published by Wiley Periodicals LLC.

**Basic Protocol 1**: Thawing, recovery, and passaging of cryopreserved hPSCs

**Support Protocol 1**: Preparation of solutions and media for hPSC maintenance and expansion

**Support Protocol 2**: Preparation of Matrigel‐coated plates for passaging hSPCs

**Basic Protocol 2**: Differentiation of hPSCs to SC‐islets

## INTRODUCTION

Many people with type 1 diabetes (T1D) must constantly and diligently manage their blood glucose levels with exogenous insulin injections. Though effective, this treatment can also have serious adverse impacts on quality of life, is often prohibitively expensive, and requires constant and careful attention. A long‐term goal of researchers in the T1D field has been to develop improved therapies for people with T1D that restore their insulin independence, alleviating the need for complicated and rigorous insulin therapies. This therapeutic goal gained momentum in the 1970s when a rat‐derived endocrine tissue (pancreatic islet) transplant successfully alleviated hyperglycemia in diabetic rodents (Ballinger & Lacy, 1972; Kemp et al., 1973). Since then, researchers have pursued islet transplantations to similarly treat patients with T1D using human islet transplantation protocols that were refined in the late 1990s by the Shapiro group and remain the gold standard for islet replacement therapies (Shapiro et al., 2000). Despite the success of islet transplantation, these protocols still face major challenges, such as donor scarcity and complications resulting from the immunosuppression that is inherently required for this treatment (Latres et al., 2019; Scharp & Marchetti, 2014; Shapiro et al., 2006). One potential solution to these problems is to generate in vitro‐derived islets from hPSCs using a stepwise differentiation protocol that is largely informed by natural development. These SC‐islets would provide a renewable and unlimited source of insulin‐secreting β‐cells, replacing the need for donor islets. In fact, several ongoing clinical trials using SC‐islets have provided exciting results illustrating the utility of these cells (Agulnick et al., 2015; Pullen, 2022; Wang et al., 2024). One common drawback associated with these studies, however, is that SC‐islets are immature compared to native human islets. Many groups have focused on improving and optimizing SC‐islet protocols to make them more glucose responsive and increase their functional maturity, but thus far there has been no “silver bullet” that drastically improves their function, and it remains challenging to perfectly reproduce protocols derived by different groups (Balboa et al., 2022; Barsby et al., 2022; Hogrebe et al., 2021; Nair et al., 2019; Pollock et al., 2023; Sali et al., 2025).

A major goal in the SC‐islet field is to generate clusters of cells that are as similar as possible to native islets of Langerhans. Islets reside in the pancreas and comprise different cell types, including α‐, β‐, and δ‐cells, which release hormones such as glucagon, insulin, and somatostatin, respectively, to maintain glucose homeostasis. To mimic this vital function of islets, SC‐islet protocols aim to recapitulate in vivo development in a dish by guiding undifferentiated, pluripotent stem cells (PSCs) through the various stages of pancreas specification using signals to induce each step of development, with the goal of producing functional islets (Bandral et al., 2025; Lorberbaum et al., 2020).

Advances in the engineering of embryonic and induced pluripotent stem cells have been vital to the development of these therapies, as recapitulating development in a dish is not an easy feat (Clark et al., 2004; Takahashi et al., 2007; Takahashi & Yamanaka, 2006). Some of the first SC‐islets were described in 2006 (D'Amour et al., 2006), leading to SC‐islet transplants that were able to return hyperglycemic diabetic mice to a euglycemic state (Rezania et al., 2014). A major goal in the field is to develop an efficient and repeatable directed islet differentiation protocol that functions in all hPSC genetic backgrounds. Clinical trials of SC‐islet transplantations have been in the works for more than a decade, highlighting their therapeutic potential (Agulnick et al., 2015; Pullen, 2022; Wang et al., 2024). Simultaneously, many groups have been working to optimize their protocols. For example, improved glucose responsiveness was achieved by focusing on β‐cell maturation through suspension and well plate shape (Balboa et al., 2022), and Braam et al. demonstrated the generation of stem‐cell‐derived pancreatic progenitors to SC‐islets using a commercially available kit, making this technology more accessible while also highlighting that these SC‐islets had lower glucose responsiveness than primary human islets (Braam et al., 2023). Whether the differentiation protocol is an “in‐house” recipe or is commercially available, optimization is still necessary to reach the goal of making SC‐islets clinically available for patients with T1D.

A fine‐tuned, scalable, and consistent method to generate these endocrine cells in a dish would greatly improve T1D‐directed therapies but also provide an important platform to reliably define novel mechanisms and clarify the partially understood mechanisms of pancreas development. For instance, many groups use stem cells to study patient‐specific mutations, test compounds on hPSCs, and study various pancreas pathologies. Findings from different differentiation protocols, however, can vary from one group to another. Although the general scheme of all differentiations is similar, there are often differences in protocols, and many recipes are proprietary. Despite these challenges, the goal of our work here is to contribute to overcoming the challenge of reproducibility by sharing our protocols for generating SC‐islets. We include in‐depth descriptions starting with the basics of thawing hESCs/hPSCs and describe culturing conditions that result in high‐quality human SC‐islets, including checkpoints used to assess the efficiency of the procedure. We have taken special care to ensure that these protocols are accessible and reproducible for all trainees interested in SC‐islet differentiations.

## STRATEGIC PLANNING

**Figure 1:**
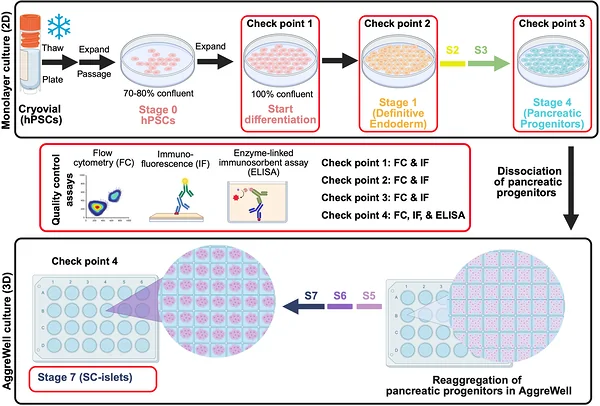
Overview of the process for generating SC‐islets derived from human pluripotent stem cells (hPSCs).

These protocols provide a detailed, step‐by‐step guide to differentiating human pluripotent stem cells (hPSCs), including human embryonic stem cells (hESCs) or human induced pluripotent stem cells (hiPSCs), into SC‐islets through a series of seven stages that mimic pancreas development (Fig. 1). Using this protocol, hPSCs can be differentiated into SC‐islets in ∼43 days, and because the procedure is long, laborious, and technically demanding, careful attention to detail is essential. These detailed protocols are designed to help researchers navigate and troubleshoot common challenges that occur during the generation of SC‐islets from hPSCs.

## Basic Protocol 1 THAWING, RECOVERY, AND PASSAGING OF CRYOPRESERVED hPSCs

Careful handling during thawing and recovery of hPSCs is important to maintain viability and pluripotency. Because cryopreservation is highly stressful to the cells, the initial 24‐48 hr after thawing are critical for their successful attachment and survival. This protocol presents a reliable, stepwise method for appropriately thawing cryopreserved hPSCs that results in optimal growth for downstream applications. Thawing of cryopreserved hPSCs should be performed rapidly and in the cell culture biosafety cabinet to maximize cell viability and sterility.

### Material

Matrigel (hPS qualified; cat. no. 345277, Corning)
DMEM/F‐12 (cat. no. 21331020, Gibco), 37°C and 25°C
hPSC cell lines, stored frozen in liquid nitrogen
70% (v/v) ethanol
Y‐27632 ROCK inhibitor cat. no. ab120129, Abcam)
Complete mTeSR1 medium (see Support Protocol 1)
Dulbecco's phosphate‐buffered saline (DPBS), no calcium, no magnesium (cat. no. 14190‐250, Gibco)
ReLeSR (cat. no. 100‐0483, Stem Cell Technologies)
Pen/Strep (cat. no. 15140, Gibco)

Laminar flow hood/biosafety cabinet
6‐well plates (cat. no. 3516, Corning)
Water bath, 37°C
50‐ and 50‐ml conical tubes (cat. nos. 653 PG and 618 PG, Dot Scientific)
37°C, 5% CO_2_ incubator
1000‐µl (P1000) pipet and appropriate tips
150‐ml vacuum system (cat. no. 431153, Corning)

#### Thawing and recovery of cryopreserved hPSCs

**Figure 2:**
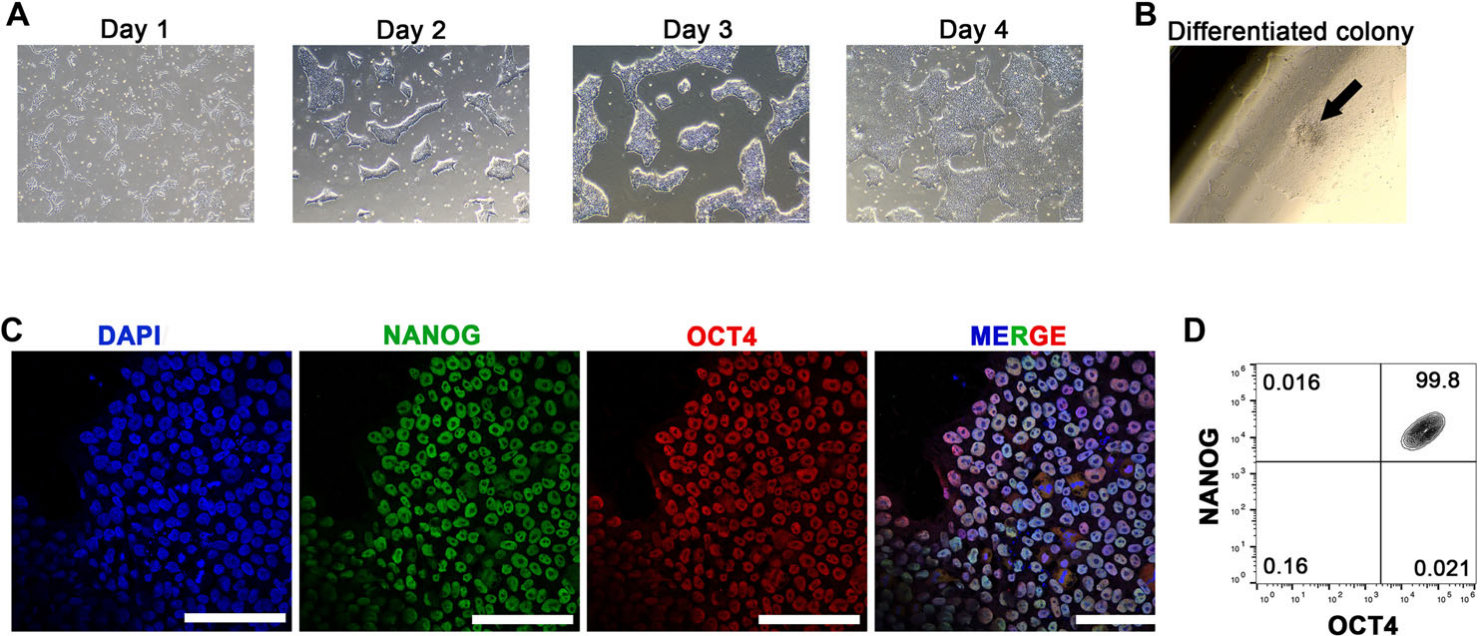
Culturing human pluripotent stem cells. (**A**) Representative brightfield images showing the morphology of human pluripotent stem cells (hPSCs). (**B**) Brightfield image highlighting (arrow) the morphology of spontaneously differentiated hPSC colony. (**C**) Immunofluorescence and (**D**) flow cytometry (FC) analyses (*n* = 3 independent experiments) confirm robust expression of pluripotency markers (OCT3/4 and NANOG). Scale bars, 100 µm.

1 At least 1 hr before thawing the cryopreserved hPSCs to begin the protocol, prepare a 6‐well plate coated with 1:50 Matrigel (see Support Protocol 2).
2 Add 4 ml prewarmed (37°C) DMEM/F‐12 to a 15‐ml conical tube.
3 Remove a cryovial of hPSCs from liquid nitrogen chamber and thaw it by gently agitating it in a prewarmed 37°C water bath until you see only small ice crystals remaining.
  This should not take more than 1‐2 min.
4 Dry the cryovial, clean the outside with 70% ethanol, and then move it to a laminar flow hood before continuing.
5 Using a 1000‐µl (P1000) pipet, transfer the cells in a dropwise manner (to preserve the aggregates of hPSCs from the cryovial) to 4 ml prewarmed DMEM/F‐12 medium supplemented with 10 µM Y‐27632 just before use.
  Attention should be paid to avoid excessive pipetting, as this can disrupt the integrity of the cell aggregates.
6 Centrifuge the cell suspension for 4 min at 600 × *g*, room temperature.
7 Carefully remove the supernatant and gently resuspend the pellet in 2 ml mTeSR1 medium containing 10 µM Y‐27632.
8 Aspirate the 1:50 Matrigel from the wells of the plate from step 1, and wash it by adding 2 ml/well DMEM/F‐12 and then aspirating the DMEM/F‐12.
9 Aspirate the DMEM/F‐12 and transfer the cell suspension to one well of the 6‐well plate. Place the plate in a 37°C, 5% CO_2_ incubator and incubate overnight.
  It is common to observe significant cell death on the day after thawing cells.
10 The next morning, aspirate the medium to remove dead cells and debris and wash the wells with 2 ml DPBS, aspirating after the wash.
11 Add 2 ml/well complete mTeSR1 medium (without Y‐27632), prewarmed at 25°C. Culture cells in a 37°C, 5% CO_2_ incubator, performing daily medium changes with mTeSR (without Y‐27632) and visually assessing the cells each day to monitor morphology and confluency.
  It is common to observe significant cell death next day after thaw the cells.
  Cells are usually ready for passaging within 3‐4 days after being thawed (depending on how many cells were thawed), as indicated by the appearance of packed, well‐distributed undifferentiated colonies (Fig. 2A).
  If only a few colonies are observed after thawing, it may be necessary to transfer the cells into a smaller well format without splitting. However, in cases of poor recovery, it is better to thaw a fresh vial if available.

#### Maintenance and expansion of hPSCs

hPSCs should be rigorously maintained to preserve pluripotent and undifferentiated cells before robust expansion to achieve high‐quality and reproducible differentiation results. Any signs of stress, spontaneous differentiation, or suboptimal confluency at the start of differentiation can significantly compromise the efficiency of desired lineage specification and downstream maturation. Therefore, hPSCs must be carefully maintained to reach the appropriate density and handled using standardized passaging methods. Here, we present a protocol outlining all the steps required to ensure hPSC quality, with detailed explanations of routine maintenance and expansion, which all are essential to prepare high‐quality cells for SC‐islet differentiation.

#### Passaging of hSPCs

Before the start of each differentiation, hPSCs should be expanded to the appropriate number required to start the differentiation. Below are the instructions for expanding hPSCs plated onto hES‐qualified Matrigel from one well of a 6‐well plate.

12 At least 1 hr before passaging, coat the wells of a 6‐well plate with 1:50 Matrigel (see Support Protocol 2) and incubate for 1 hr at room temperature.
13 During the incubation, prepare the required volumes of mTeSR1, ReLeSR, and DMEM/F‐12 as described in Support Protocol 1, and keep warm at 25°C.
14 Use a microscope to visually scan the complete well to identify regions of spontaneous differentiation (patchy appearance within the colony; Fig. 2B), if there are any. The development of such regions in the culture should be avoided by performing daily medium change and preventing over‐confluency.
15 Swirl the plate, aspirate mTeSR from the well, and add 2 ml/well DPBS to wash the cells.
16 Swirl the plate and aspirate the DPBS. Add 1 ml prewarmed (25°C) ReLeSR and incubate for 1 min in the hood and aspirate it after 1 minute.
17 Incubate the plate without any liquid at 37°C for 1‐2 min (depending upon the degree of confluency).
  If spontaneously differentiated cells are present in the culture (Fig. 2B), incubation time should be restricted to 1 min to prevent their detachment.
18 Add 1 ml prewarmed mTeSR and firmly tap the plate from the bottom and sideways for ∼30 sec Most of the colonies should be detached by now.
19 Detach the remaining colonies with a P1000 pipet, gently pipetting to generate a homogenous suspension of aggregates containing ∼3‐5 cells each. Avoid generating a single‐cell suspension by overpipetting. Larger cell aggregates during passaging will increase the chances of spontaneous differentiation in subsequent cultures.
20 For wells at 70% confluence before aggregate formation, the cultures should be split at a 1:6 ratio, which typically yields a 70‐80% confluent culture within 3‐4 days (Fig. 2A, day 4).
  The splitting ratio should be adjusted according to the doubling rate of cell type being used and will vary among cell lines and culture plates, including culture flasks.
21 After passaging the cells, plate them into a 6‐well plate in 2 ml mTeSR medium supplemented with 10 µM/well Y‐27632.
22 Place the plate in a 37°C, 5% CO_2_ incubator. Perform medium change early the next morning; this will also remove dead cells, which are normal.
23 Perform daily medium changes with mTeSR, including a 2‐ml DPBS wash as above with every change. Visually assess cultures to monitor growth until the next passaging time.
  Cells should be ready for differentiation within 3‐4 days after being split; therefore, the split ratio must be adjusted to ensure that they reach the appropriate confluency in this time frame. If they grow too slowly or too quickly, change the seeding density accordingly.
  After hPSC passaging, it is important to confirm that the cells retain their pluripotent state during their maintenance. Evaluating pluripotency ensures the success of subsequent differentiations. At this point, hPSCs should be packed in compact colonies with defined edges, and any indication of spontaneous differentiation (Fig. 2B) or heterogeneous morphology can compromise the efficiency and reproducibility of differentiation. hPSCs should be enriched in the expression of pluripotency markers such as OCT3/4 and NANOG. Therefore, validating pluripotency through immunostaining (Fig. 2C) or flow cytometry (Fig. 2D) at this stage serves as a critical quality‐control checkpoint.

## Support Protocol 1 PREPARATION OF SOLUTIONS AND MEDIA FOR hPSC MAINTENANCE AND EXPANSION

### Materials

mTeSR (cat. no. 05850, Stem Cell Technologies)
Pen/Strep (cat. no. 15140, Gibco)
Matrigel, 1:30 and 1:50 dilutions (see Support Protocol 2)
ReLeSR (cat. no. 100‐0483, Stem Cell Technologies)
Dulbecco's phosphate‐buffered saline (DPBS), no calcium, no magnesium (cat. no. 14190‐250, Gibco)
2% bovine serum albumin (BSA; fatty acid free, low endotoxin) in MCDB 131, no glutamine (cat. no. 10372‐019, Gibco)
TrypLE (cat. no. 12604013, Gibco)

6‐well plates (cat. no. 3516, Corning)
Water bath, 25°C

#### Preparation of complete mTeSR medium—timing: 30 min once mTeSR is thawed

1 Pre‐thaw the 5× mTeSR supplement overnight at 4°C to prepare the amount of complete mTeSR medium required for the desired number of wells.
  After thawing, 5× supplement can be aliquoted and stored at –20°C for future use.
2 Before use, mix 30 ml of thawed 5× supplement with 120 ml of basal mTeSR medium.
3 Add 100× Pen/Strep to the medium to 1× concentration (e.g., 1.5 ml for 150 ml of medium).
  This complete mTeSR medium can be stored at 4°C for up to 2 weeks.
  Throughout the protocol, each well of a 6‐well plate requires 2 ml of medium change.

#### Preparation of reagents and 6‐well plate for hPSC passaging—timing: 30 min

4 Homogeneously coat the wells of a 6‐well plate with a 1:50 dilution of Matrigel stock (see Support Protocol 1).
5 Aliquot appropriate amounts of complete mTeSR (2 ml/well) and ReLeSR (1 ml/well) and warm plate at 25°C for 30 min.
  ReLeSR is a nonenzymatic solution, which helps the colonies to detach easily.
6 Aliquot 2 ml/well DPBS and warm it 25°C for 30 min.

#### Preparation of reagents and medium for hPSC differentiation—timing: 30 min

7 Homogeneously coat the wells of a 6‐well plate with a 1:30 dilution of Matrigel stock.
8 Aliquot the needed amounts of complete mTeSR medium (see steps 1‐4), 2% BSA in MCDB, and TrypLE (1 ml/well) and warm them at 25°C for 30 min.
  TrypLE is an enzymatic solution that helps create a single‐cell suspension for differentiation. The 2% BSA in MCDB will help neutralize the enzyme and prevent over‐digestion of cells.
9 Aliquot the needed amount of DPBS and warm it at 25°C for 30 min.

## Support Protocol 2 PREPARATION OF MATRIGEL‐COATED PLATES FOR PASSAGING hSPCs

### Materials

Matrigel, frozen
DMEM/F‐12, 4°C and 37°C

Tubes and plates required for the procedure being performed
Laminar flow hood

#### Preparation of 1:5 Matrigel stock—timing: 10‐15 min once Matrigel is thawed

1 Pre‐thaw 5 ml Matrigel at 4°C for the coating of a plate.
  This will take 2‐3 hr.
2 Add 5 ml Matrigel to 20 ml cold (4°C) DMEM/F‐12 to achieve a 1:5 dilution and mix thoroughly.
3 Make 1 ml aliquot of 1:5 diluted Matrigel into the labeled tubes.
4 Store the aliquots at –20°C for long‐term storage.

#### Preparation of working dilutions of Matrigel from 1:5 stock—timing: 10‐15 min

Prepare working dilutions immediately before use.

5a *For passaging*:
  • Add 1 ml of 1:5 stock Matrigel to 9 ml cold DMEM/F‐12 to achieve a dilution 1:50.
  • Mix well before use.
5b *For differentiation*:
  • Add 1 ml of 1:5 stock Matrigel to 5 ml cold DMEM/F‐12 to achieve a dilution of 1:30.
  • Mix well before use.

#### Preparation of Matrigel‐coated plates for hPSC thawing, passaging, and expansion—timing: 1 hr

6 Thaw at room temperature an amount of 1:5 Matrigel stock aliquots sufficient to coat the plates needed.
7 Prepare 1:50 dilution by adding 1 ml of 1:5 Matrigel stock to 9 ml cold DMEM/F‐12.
  One preparation of the 1:50 dilution is sufficient to coat 10 wells of a 6‐well plate.
8 Keep the plates inside the laminar flow hood at room temperature for 1 hr.
9 Aspirate the Matrigel and add 2 ml DMEM/F‐12, prewarmed at 37°C, to each well of the 6‐well plates.
10 Aspirate the DMEM/F‐12 just before the start of any procedure (passaging or differentiation).

## Basic Protocol 2 DIFFERENTIATION OF hPSCs TO SC‐ISLETS

### Materials

hPSC cell lines in 6‐well plates (Basic Protocol 1)
mTeSR1 medium (cat. no. 05850, Stem Cell Technologies)
Matrigel (cat. no. 345277, Corning)
Albumin bovine fraction (BSA), fatty acid free, low endotoxin (cat. no. 11945, Serva)
MCDB 131 medium, no glutamine (cat. no. 10372‐019, Gibco)
Dulbecco's phosphate‐buffered saline (DPBS), no calcium, no magnesium (cat. no. 14190‐250, Gibco)
TrypLE EXPRESS (cat. no. 12604013, Gibco)
0.4% trypan blue (cat. no. 15250061, Gibco)
DMEM/F‐12 (cat. no. 21331020, Gibco)
Glucose (cat. no. G8769, Sigma)
Sodium bicarbonate (cat. no. S6297, Sigma)
Glutamax (cat. no. 35050038, Gibco)
Penicillin/streptomycin (Pen/Strep; cat. no. 15140, Gibco)
Insulin transferrin selenium (ITS‐X; cat. no. 51500056, Gibco)
Activin A (cat. no. 338‐AC/CF, R&D Systems)
CHIR99021 (cat. no. 4423, Tocris)
Vitamin C (ascorbic acid; cat. no. A4544‐25G, Tocris)
Human fibroblast growth factor 7 (FGF‐7; cat. no. 251‐KG‐01M/CF, R&D Systems)
IWP‐2 (cat. no. 501975884, Stem Cell Technologies)
Sant1 (cat. no. S4572, Sigma)
Retinoic acid (RA; cat. no. R2625, Sigma)
TPPB (cat. no. 5343, Sigma)
LDN193189 HCl (cat. no. NC1730738, Stem Cell Technologies)
Y‐27632 ROCK inhibitor (cat. no. ab120129, Abcam)
Human epidermal growth factor (hEGF; cat. no. AF100151MG, PeproTech)
Nicotinamide (cat. no. 72340‐100G, Sigma)

24‐well AggreWell plate (Stem Cell Technologies AggreWell 400, cat. no. 34415)
Anti‐Adherence Rinsing Solution (cat. no. 07010, Stem Cell Technologies)
DMEM/F‐12 medium (cat. no. 21331020, Gibco)
Heparin (cat. no. 2106‐10VL, Sigma)
StemMACS RepSox ALK5 inhibitor (cat. no. 14794, Cayman Chemical Company)
l‐3,3ʹ,5‐Triiodothyronine, sodium salt (cat. no. T6397, Sigma)
Zinc sulfate heptahydrate (ZnSO_4_•7H_2_O; cat. no. Z0251, Sigma)
γ‐secretase inhibitor XX, compound E (cat. no. 565789, Millipore Sigma)
Sodium pyruvate (cat. no. 11360070, Gibco)
*N*‐Acetyl‐l‐cysteine (cat. no. A9165, Sigma)
Trolox (cat. no. 9002, Tocris)
R428 (cat. no. 21523, Cayman Chemical Company)
Primary and secondary antibodies used in Basic Protocol 2: e.g.,
  Mouse anti‐OCT3/4 (1:50; cat. no. SC5279, Santa Cruz Biotech)
  Goat anti‐NANOG (1:40; cat. no. AF1997, R&D Systems)
  Mouse anti‐SOX17 (1:100; cat. no. MAB19241SP, R&D Systems)
  Mouse anti‐FOXA2 (1:100; cat. no. IC2400G, R&D Systems)
  Goat anti‐PDX1 (1:40; cat. no. AF2419, R&D Systems)
  Mouse anti‐NKX6.1 (1:100; cat. no. F55A10, DSHB)
  Rabbit anti‐SOX9 (1:1000; cat. no. AB5535, Millipore Sigma)
  Guinea pig anti‐insulin (1:5; cat. no. Agilent IRoo261‐2, Dako)
  Rabbit anti‐glucagon (1:100; cat. no. 2760S, Cell Signaling Technologies)
  Rabbit anti‐somatostatin (1:300; cat. no. H‐060‐03, Pheonix Pharmaceuticals)
  Rabbit anti‐MAFA (1:200; cat. no. ab26405, Abcam)
  Mouse anti‐ISL1 (1:200; cat. no. 39.4D5‐c, DSHB)
  Donkey anti‐mouse (1:500; cat. no. A10037, Invitrogen)
  Donkey anti‐goat (1:500; cat. no. A21447, Invitrogen)
  Donkey anti‐rabbit (1:500; cat. no. A21206, Invitrogen)
  Donkey anti‐guinea pig (1:500; cat. no. 706‐575‐148, Jackson ImmunoResearch)
Insulin Chemiluminescence ELISA kit (cat. no. 80‐INSHU‐CH01, ALPCO)

Laminar flow hood
6‐well plate (cat. no. 3516, Corning)
50‐ and 15‐ml conical tubes (cat. nos. 653 PG and 618 PG, Dot Scientific)
150‐ml vacuum system (cat. no. 431153, Corning)
37°C, 5% CO_2_ incubator
1000‐µl (P1000) pipet and appropriate tips
40‐µm Pluristrainer (cat. no. 431004060, Pluriselect)
Invitrogen automated cell counter (Countess; cat. no. AMQAF2000, ThermoFisher Scientific)
40‐µm‐pore‐size cell strainer (cat. no. 087711, Falcon)
Single‐channel VIAFLO (cat. no. 4014, Integra)

#### Preparation of Cells for Differentiation—Timing: 2 Days

**Figure 3:**
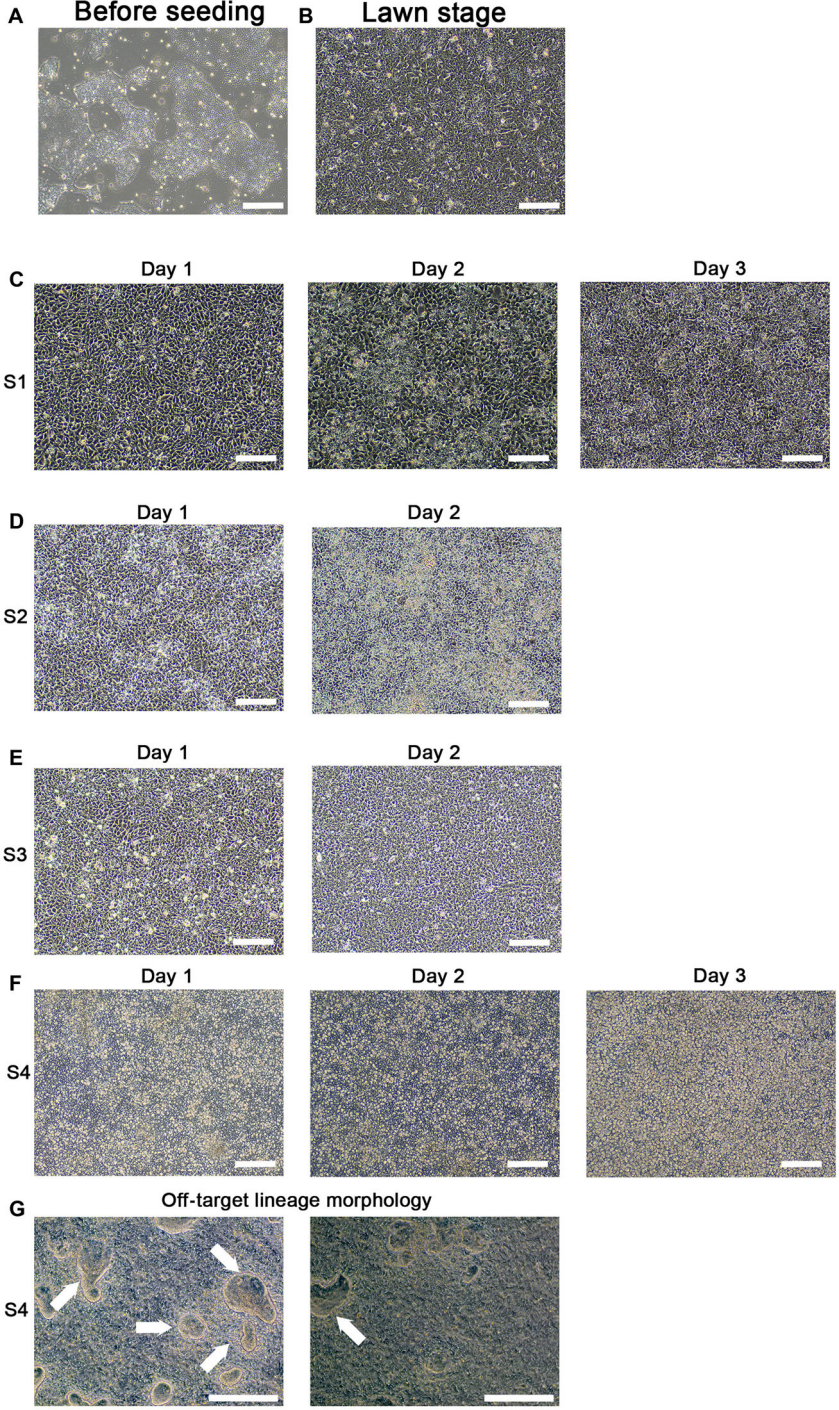
Early morphological changes illustrate the stepwise differentiation of hPSCs through pancreatic progenitors. (**A**) Confluency of hPSCs at the start of seeding. (**B**) Compact morphology of hPSCs at the start of differentiation. (**C**) Representative brightfield images of definitive endoderm (S1) on Days 1, 2, and 3. (**D**) Brightfield images showing primitive gut tube specification (S2) on the indicated days. (**E**) Representative brightfield images illustrating morphological changes during the posterior foregut stage (S3) on the indicated days. (**F**) Representative brightfield images depicting the induction of pancreatic progenitors (S4) with epithelial morphology. (**G**) Brightfield images showing examples of off‐target morphology (white arrows) at the end of S4. Scale bars, 100 µm.

1 In the morning, at least 4‐6 hr before starting the differentiation, add fresh mTeSR medium to the cells in the 6‐well plates.
  Cells should be 70‐80% confluent, and colonies should not show any signs of differentiation (Fig. 3A).

2 At least 1 hr before the start of differentiation, coat fresh plates with hES‐qualified Matrigel at a 1:30 dilution (see Support Protocol 2).
3 Prepare appropriately sized aliquots (2 ml/well) of mTeSR medium, 2% BSA in MCDB, and TrypLE Express (see Support Protocol 1), and warm them at 25°C before beginning the cell dissociation process.
4 Swirl the plate and aspirate the medium from the wells.
5 Wash once with 2 ml/well DPBS and aspirate the DPBS.
6 Add 1 ml prewarmed TrypLE Express to the appropriate well(s).
7 Incubate the plates at 37°C for 3‐4 min (can vary between cell lines) and check cells under a microscope for dissociation.
8 First tap the plate sideways as soon as cells round up and then, using a P1000, pipet up and down four or five times to achieve a single‐cell suspension (SCS).
9 After generating SCS, add 1 ml/well of 2% BSA in MCDB to neutralize the enzyme and mix well. Filter the cell suspension through a 40‐µm Pluristrainer (to ensure separation into single cells).
10 Transfer the filtered single‐cell suspension to a 15‐ml conical tube using a serological pipet and spin for 4 min at 400 × *g*, 25°C.
11 Aspirate the supernatant without disturbing the pellet. Add 1 ml prewarmed mTesR1 and resuspend using a P1000 to gently dispersing the cell pellet.
12 Using an automatic cell counter, count the cells to achieve sufficient cells required for the seeding of differentiation. For example: Take 15 µl of the cell suspension, add 15 µl of 0.4% trypan blue, and mix well.
  Always take two independent counts and average for better accuracy. If count values vary by more than 10%, repeat the count to attain appropriate seeding number.
  Cell viability should be ≥90%. If survival is <90%, the single‐cell suspension is suboptimal.
13 Seed 2.5 million live cells per well into the 6‐well plate. The total volume of medium should be 2 ml per well. Add 10 µM Y‐27632 to each well and swirl the plate to evenly distribute the cells in each well.
14 Place the plate in a 37°C, 5% CO_2_ incubator overnight.
15 The next morning, wash the wells with 2 ml DPBS and aspirate it from of the wells.
16 Add 2 ml prewarmed mTeSR and incubate at 37°C, 5% CO_2_ for 6 hr before the start of your planned differentiation.
  After the 6 hr of incubation, hPSCs should look like a lawn of cells (Fig. 3B).

#### Differentiation of hPSCs to Pancreatic Progenitor (PP) Stage (Stages S1‐S4)

*NOTE*: During the entire procedure from Stage 1 to Stage 4, cells should be gently washed with 2 ml DPBS before a medium change every day. This ensures the removal of dead cells. For more details, refer to Figure 3.

#### Stage 1 (S1): Definitive endoderm—timing: 3 days

Brightfield pictures in Figure 3C (S1) can be used as reference images for all 3 days of S1.

##### Day 1 (S1D1)

17 Prepare the required volume of basal medium for Stages 1 and 2 (S1/2 basal medium; Table 1), filter sterilize, and store at 4°C.

**Table 1:** Basal Components of Stage 1/2 Medium

Reagent	Final concentration in medium
MCDB medium	Same as stock
Glucose	2.5 mM
NaHCO_3_	1.5 g/liter
BSA	0.5%
Glutamax	1%
Penicillin/streptomycin	1%
Insulin‐transferrin selenium	0.005%

18 Prepare fresh complete S1D1 medium by adding supplements (Table 2) to S1/2 basal medium just before feeding. Warm the medium at 37°C for 15 min.

**Table 2:** Medium Supplements for Stage 1

Stage 1 (3 days)	Reagent	Final concentration in medium
Day 1	Activin A	100 ng/ml
	CHIR	3 µM
Day 2	Activin A	100 ng/ml
	CHIR	0.3 µM
Day 3	Activin A	100 ng/ml
	Vitamin C	0.25 mM
	FGF‐7	50 ng/ml

19 Gently aspirate the old mTeSR from the hPSC plates (from step 16).
20 Wash with 2 ml DPBS and aspirate the DPBS.
21 Add 2 ml/well prewarmed (37°C) S1D1 medium and incubate at 37°C for 24 hr.
  It is common to see dead cells in the culture the next morning.

###### Day 2 (S1D2)

22 Prepare fresh medium by adding supplements (Table 2) to the S1/2 basal medium, and warm it at 37°C for 15 min.
23 Aspirate the old medium, wash once with 2 ml DPBS, and aspirate the DPBS.
24 Add 2 ml/well prewarmed S1D2 medium to plates and incubate at 37°C for 24 hr.

###### Day 3 (S1D3)

**Figure 4:**
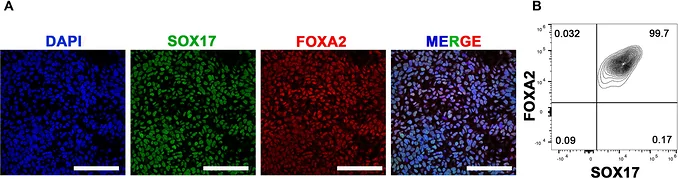
High induction of definitive endoderm (S1) lineage. (**A**) Immunofluorescence and (**B**) flow cytometry (FC) analyses (*n* = 3 independent experiments) highlighting high induction of DE markers (SOX17 and FOXA2). Immunofluorescence images are representative merged confocal *z*‐stacks; scale bars, 100 µm.

25 Prepare fresh medium by adding supplements (Table 2) to the S1/2 basal medium and warm it at 37°C for 15 min.
26 Aspirate the old medium, wash once with DPBS, and aspirate the DPBS.
27 Add 2 ml/well prewarmed S1D3 medium and incubate at 37°C for 24 hr.
  To assess the induction efficiency of the definitive endoderm (S1) stage, immunofluorescence (Fig. 4A) and flow cytometry (Fig. 4B) can be used to evaluate key markers of definitive endoderm (DE), such as FOXA2 and SOX17. High, homogeneous expression of FOXA2 and SOX17 show robust induction of this stage.

#### Stage 2 (S2): Primitive gut tube—timing: 2 days

Brightfield pictures in Figure 3D (S2) can be used as reference images for both days of S2.

##### Day 1 (S2D1)

28 Using the same Stage 1/2 basal medium, add the three Stage 2 supplements (Table 3) and mix well.
  Prepare sufficient complete S2 medium for both days

**Table 3:** Medium Supplements for Stage 2

Stage 2 (2 days)	Reagent	Final concentration in medium
Days 1+2	Vitamin C	0.25 mM
	FGF‐7	50 ng/ml
	IWP‐2	1.25 µM

29 Aspirate the old medium, wash once with 2 ml DPBS, add 2 ml/well prewarmed Stage 2 medium, and incubate at 37°C for 24 hr.

###### Day 2 (S2D2)

30 Repeat step 29
  At the end of S2D2, small 3D aggregates may appear occasionally; vigorously shake the plate during the medium changes in the subsequent stages to ensure the proper removal these structures. Differentiating cells should be well adhered to the plate so you should not lose excess cells.

#### Stage 3 (S3): Posterior foregut—timing: 2 days

Brightfield pictures in Figure 3E (S3) can be used as reference images for both days of S3.

Prepare the required volume of basal medium for both Stages 3 and 4 (Table 4), filter sterilize, and store at 4°C. The medium composition is the same for both days of S3.

**Table 4:** Basal Components of Stage 3/4 Medium

Reagent	Final concentration in medium
MCDB medium	Same as stock
Glucose	4.5 mM
NaHCO_3_	2.4 g/liter
BSA	2%
Glutamax	1%
Penicillin/streptomycin	1%
Insulin‐transferrin selenium	0.5%

##### Day 1 (S3D1)

31 Supplement the S3/4 basal medium (Table 4) with the five S3 supplements (Table 5) and mix well. Prepare complete S3 medium sufficient for both days

**Table 5:** Medium Supplements for Stage 3

Stage 3 (2 days)	Reagent	Final concentration in medium
Days 1+2	Vitamin C	0.25 mM
	FGF‐7	50 ng/ml
	Sant1	0.25 µM
	Retinoic acid	1 µM
	TPPB	200 nM
	LDN193189 HCl	100 nM

32 Aspirate the old medium, wash once with 2 ml DPBS, add 2 ml/well prewarmed S3 medium, and incubate at 37°C for 24 hr.

33 Repeat step 32 for S3D2 and S3D3, respectively.
  If small 3D aggregates are still present, shake the plate again, as in S2, during medium changes. The desired cells should remain attached.

#### Stage 4 (S4): Pancreatic progenitors (PP)—timing: 4 days (3 days in monolayer and 1 day in AggreWell)

Brightfield pictures in Figure 3F (S4) can be used as reference images for both days of S4.

##### Day 1 (S4D1)

34 Add the nine S4 supplements (Table 6) to S3/4 basal medium (Table 4) and mix well. Prepare complete S4 medium sufficient for 4 days.

**Table 6:** Medium Supplements for Stage 4

Stage 4 (3‐4 days)	Reagent	Final concentration in medium
Days 1‐4	Vitamin C	0.25 mM
	Y‐27632	10 µM
	hEGF	100 ng/ml
	FGF‐7	2 ng/ml
	Sant1	0.25 µM
	Retinoic acid	0.1 µM
	LDN193189 HCl	200 nM
	Nicotinamide	10 mM
	TPPB	100 nM

35 Aspirate the old medium, wash once with 2 ml DPBS, add 2 ml/well prewarmed S4 medium, and incubate at 37°C for 24 hr.
  After 24 hr of S4D1, the cells start exhibiting pancreatic‐epithelium‐like structure morphology (Fig. 3F). The number of these structures increase during the subsequent days of S4. The presence of these structures indicates the expected morphology of PP cells obtained using this protocol.

###### Days 2 and 3 (S4D2 and S4D3)

**Figure 5:**
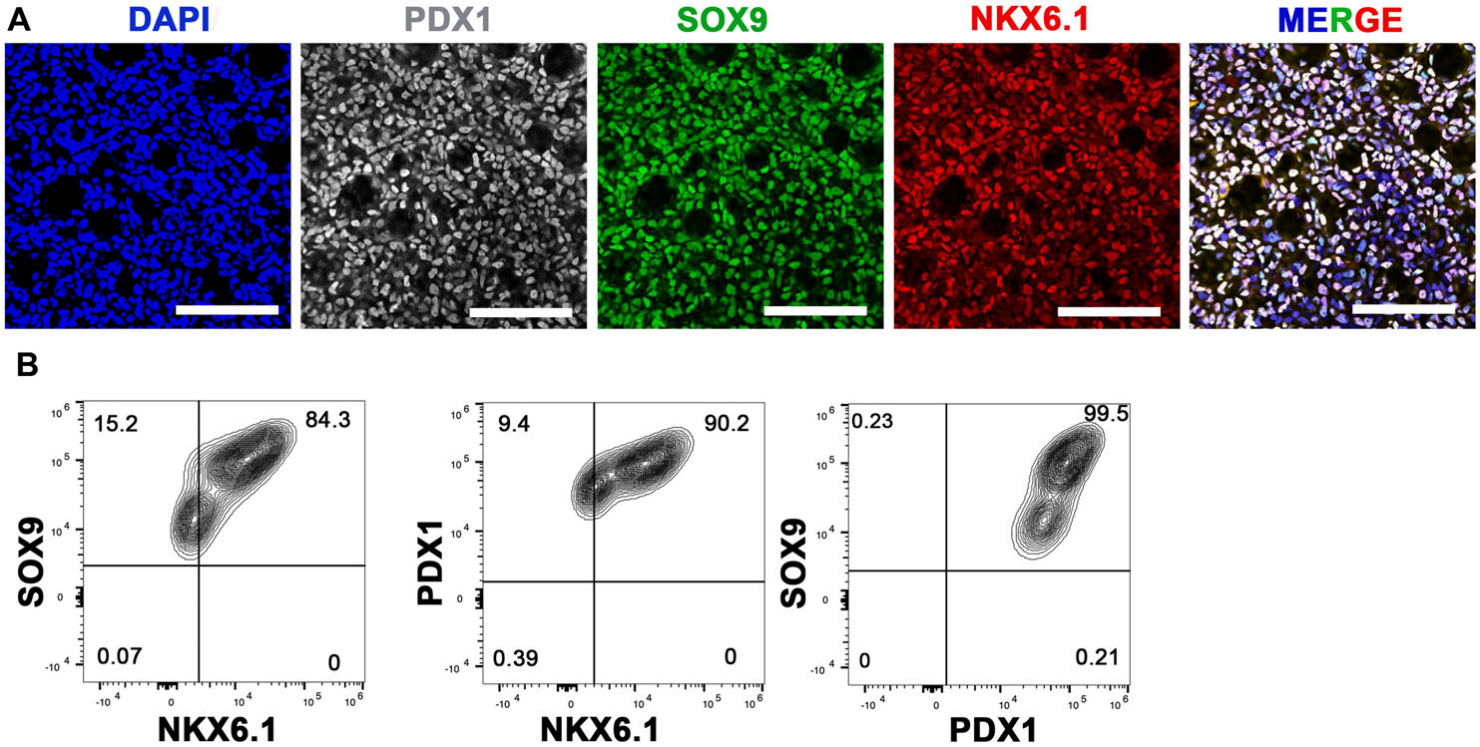
Robust and uniform generation of pancreatic progenitor (S4). (**A**) Immunofluorescence and flow cytometry analyses (*n* = 5‐7 independent experiments). (**B**) Analysis showing the robust expression of key pancreatic progenitor markers (SOX9, PDX1, and NKX6.1) on day 11. Immunofluorescence images are representative merged confocal *z*‐stacks; scale bars, 100 µm.

36 Repeat step 35 for S4D2 and S4D3, respectively
  The S4D3 stage serves as a good checkpoint for quality control (QC) analysis from the planar culture following aggregation to evaluate the quality of PPs. This can be done using immunostaining (Fig. 5A) and flow cytometry (Fig. 5B) for key markers of pancreatic progenitor markers, such as PDX1, SOX9, and NKX6.1. Performing QC at this stage helps identify suboptimal cultures early and ensures that only high‐quality differentiations are carried forward for forming SC‐islets. The timing for harvesting and collecting PPs for the QC analysis is explained in steps 40‐49 below.

#### Differentiation of Pancreatic Progenitors to SC‐Islets Using AggreWell Plate (S5‐S7—Timing: 24 Days)

**Figure 6:**
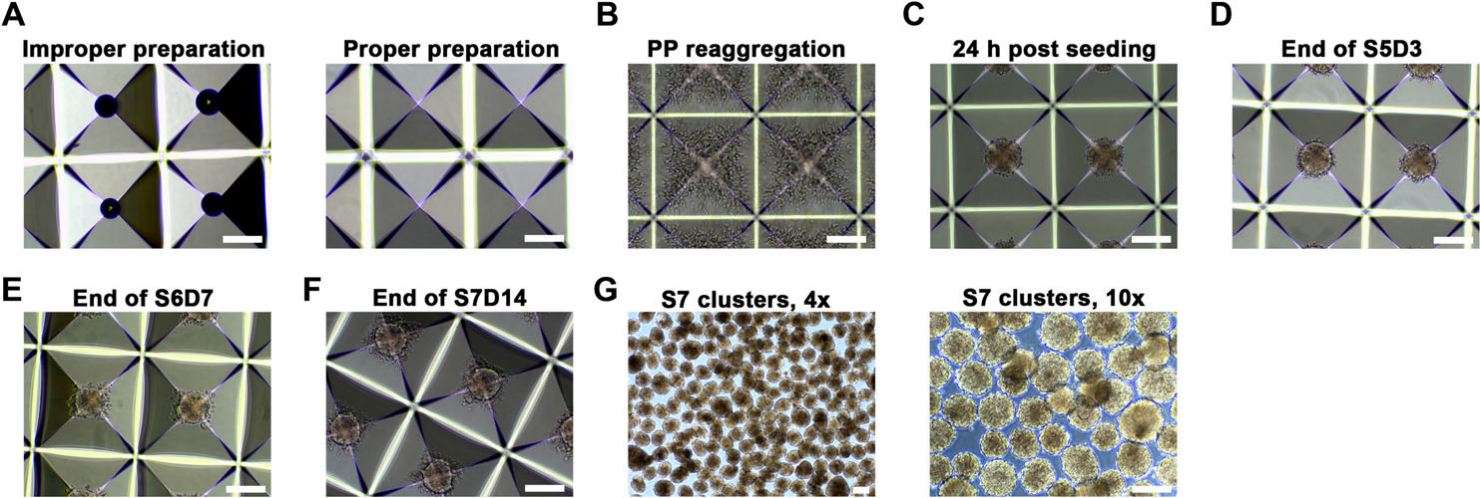
Plate preparation, seeding of pancreatic progenitors (PPs), and stage‐wise cluster morphology in AggreWell plates. (**A**) Microwells showing proper and improper examples of plate preparation before seeding (**B**) PPs evenly distributed upon reaggregation. (**C**) Homogeneous clusters formed 24 hr after reaggregation. (**D‐F**) Representative images of an AggreWell plate showing clusters at the end of S5, S6, and S7 with cell debris. (**G**) Representative brightfield images of S7 clusters following removal from AggreWell plates and filtering, highlighting compact and homogeneous morphology. Scale bars, 200 µm.

For more details on three‐dimensional differentiation, refer to Figure 6A‐F.

#### Preparation of AggreWell plates

37 Before dissociating PP cells for aggregation, prepare the 24‐well AggreWell plate as follows: Slowly add 0.5 ml/well of Anti‐Adherence Rinsing Solution to the plate; avoid creating bubbles. Keep the plate at room temperature for 4 min.
38 Visualize the plate under the microscope to observe any possible bubble formation (Fig. 6A). If bubbles are present, use a P1000 to carefully pipet up and down to remove them.
39 Once the well is bubble‐free, aspirate the anti‐adherence rinsing solution from the wells.
40 Wash each well of the AggreWell plate that you plan to use with 2 ml DMEM/F‐12, and aspirate out.
41 Add 1 ml complete S4 medium (Table 6) to each well of the 24‐well AggreWell plate that you will use.
  Although each well should be used only once, you do not have to use every well in the plate for each differentiation. Simply mark the wells in use and save the remaining wells for future differentiations.
42 Keep the plate in the 37°C incubator while you dissociate the PP cells.

#### Dissociation of PP cells

43 At the end of S4D3, aspirate the medium, wash once with 2 ml DPBS, and aspirate again.
44 Add 1 ml/well prewarmed TrypLE and incubate for ∼3 min at 37°C.
45 After 4‐5 min, observe the wells under the microscope and proceed once cells start rounding up. Dissociate the cells using a P1000 and pipet the TrypLE on top of the cells.
46 Once the single‐cell suspension is generated, add 1 ml/well 2% BSA in MCDB to inactivate the enzymes, and pipet up and down two or three times to ensure a single‐cell suspension.
47 Filter the cell suspension using a 40‐µm‐pore‐size filter. Transfer the resulting single‐cell suspension to a 15‐ml tube and centrifuge for 4 min at 600 × *g*, 25°C
48 Aspirate the medium from the tube, being careful not to disturb the pellet. Resuspend the pellet in 1 ml prewarmed complete S4 medium.
49 Using an automatic cell counter, count the PP cells to ensure that you have enough for QC and seeding of clusters.
  At this stage, an aliquot of cells can be collected for quality‐control analyses, including flow cytometry (FC) or quantitative PCR (qPCR).
50 After counting, adjust the cell density to 1.8 million cells per 0.5 ml and then add the cell suspension to one well of the 24‐well AggreWell plate previously incubated at 37°C (i.e., from step 39; Fig. 6B).
*To ensure appropriate mixing and formation of clusters, expel cells from the P1000 four times in four separate locations, moving the tip clockwise around the inside of the well, to mix and then once in the middle. This will generate clusters of homogenous size without a centrifugation step*.
51 After mixing, keep the plate in the laminar flow hood for 30 min and then transfer it to a 37°C, 5% CO_2_ incubator. Incubate for 24 hr.
52 Seeded PPs will start to aggregate and should form clusters of uniform size morphology (Fig. 6C). After 24 hr, there are expected to be very few unaggregated cells. Medium changes should be performed carefully to avoid loss of clusters in the AggreWell plate.
  We use Integra's VIAFLO Pipette to perform medium changes in AggreWell plates without disturbing the clusters.
  From this point onward, clusters require careful handling through a series of medium changes.

#### Pancreatic endocrine progenitor (S5) stage—timing: 3 days

53 Prepare complete S5 medium by supplementing the S5/6 basal medium (Table 7) with the six S5 supplements (Table 8). Prepare sufficient complete S5 medium for all three days of S5.

**Table 7:** Basal Components of Stage 5/6 Medium

Reagent	Final concentration in medium
MCDB medium	Same as stock
Glucose	14.5 mM
NaHCO_3_	1.5 g/liter
BSA	2%
Glutamax	1%
Penicillin/streptomycin	1%
Insulin‐transferrin selenium	0.5%
Heparin	1.8 U/ml

**Table 8:** Medium Supplements for Stage 5

Stage 5 (3 days)	Reagent	Final concentration in medium
Days 1‐3	Sant1	0.25 µM
	Retinoic acid	0.05 µM
	LDN193189 HCl	100 nM
	ALK5 inhibitor II	10 µM
	Triiodothyronine	1 µM
	ZnSO_4_•7H_2_O	10 µM

54 After 24 hr of incubation, carefully remove the AggreWell plate from the incubator.
55 On S5D1, replace the 1.5 ml of complete S4 medium with complete S5 medium, prewarmed at 37°C, using an Integra VIAFLO pipette on gentle/slow settings. A standard P1000 can also be used, but pipet more gently than usual so as not to disturb clusters in the microwells.
56 On S5D2 and S5D3, replace 1 ml only of the medium with fresh complete S5 medium using an Integra VIAFLO pipette.
  At the end of S5D3, clusters should look like those in Figure 6D.

#### Immature SC‐islet (S6) stage—timing: 7 days

57 Prepare complete S6 medium by adding the five S6 supplements (Table 9) to the S5/S6 basal medium (Table 7). Prepare the medium in two batches: one for the first 3 days and a second for the following 4 days.

**Table 9:** Medium Supplements for Stage 6

Stage 6 (7 days)	Reagent	Final concentration in medium
Days 1‐7	LDN193189 HCl	100 nM
	γ‐secretase inhibitor XX	100 nM
	ALK5 inhibitor II	10 µM
	Triiodothyronine	1 µM
	ZnSO_4_•7H_2_O	10 µM

58 On Day 1 (S6D1), replace 1 ml only of the previous medium with complete S6 medium, prewarmed at 37°C, using Integra's VIAFLO pipette.
59 On the next 6 days, replace 1 ml only of the previous medium with fresh complete S6 medium using Integra's VIAFLO pipette.
  At the end of S6D7, clusters should look like those in Figure 6E.

#### Towards maturation of SC‐islets (S7) stage—timing: 14 days

60 Prepare complete S7 medium by supplementing S7 basal medium (Table 10) with the six S7 supplements (Table 11). Prepare the medium in four batches: (a) first 3 days (b) next 4 days, (c) next 3 days, and (d) final 4 days.

**Table 10:** Basal Components of Stage 7 Medium

Reagent	Final concentration in medium
MCDB medium	Same as stock
Sodium pyruvate	0.5 mM
NaHCO_3_	1.5 g/liter
BSA	2%
Glutamax	1%
Penicillin/streptomycin	1%
Insulin‐transferrin selenium	0.5%
Heparin	1.8 U/ml

**Table 11:** Medium Supplements for Stage 7

Stage 7 (14 days)	Reagent	Final concentration in medium
Days 1‐14	*N*‐Acetyl‐l‐cysteine	1 mM
	Trolox	10 µM
	R428	2 µM
	ALK5 inhibitor II	10 µM
	Triiodothyronine	1 µM
	ZnSO_4_•7H_2_O	10 µM

61 At the start of S7, replace 1 ml of S7 medium with complete S7 medium, prewarmed at 37°C, using Integra's VIAFLO pipette.
62 For the next 13 days, replace 1 ml only of the previous medium with complete S7 medium using Integra's VIAFLO pipette once a day.
  At the end of S7D14, clusters should look like those in Figure 6F and G. Clusters of homogeneous sizes should be observable under 4×‐10× magnification, and clusters should maintain high viability.
  Clusters should be removed from the AggreWell plate for any further analysis.
  The end of S7 is a good checkpoint for QC analysis to validate endocrine lineage (7).

## COMMENTARY

### Background

Type 1 diabetes (T1D) is an autoimmune disease estimated to affect more than 9 million people worldwide (Bell & Lain, 2025). T1D is characterized by the body's attack on insulin‐secreting β‐cells, leaving patients with lifelong reliance on exogenous insulin. Without proper treatment, patients can develop severe hyperglycemia, leading to numerous co‐morbidities including cardiovascular disease, kidney disease, and neuropathy as well as diabetic ketoacidosis, another life‐threatening condition (Bell & Lain, 2025). Beyond insulin therapy, clinicians use other interventions to treat symptoms, including insulin pumps and glucose monitors coupled with dietary modifications that affect both the patients’ and their families’ daily lives. These treatments carry a risk of hypoglycemia, which increases the occurrence of cardiovascular events and death. Even with these challenges, insulin therapies are effective, but they are not curative. A long‐lasting treatment for people with T1D requires restoring insulin independence rather than routinely using exogenous insulin to treat recurrent and constant symptoms.

Restoration of insulin independence holds substantial potential and has attracted decades of research into stem cell therapies aimed at generating transplantable islets in vitro. These therapies were strongly influenced by the success of islet transplantations, which have been a source of hope for patients with severe T1D since the 2000s and were made famous by the Edmonton protocol, a pioneering study that demonstrated donated human islets could be transplanted into a patient with T1D, leading to euglycemia without exogenous insulin injections (Shapiro et al., 2006). Although this was an exceptional advancement in T1D care and remains the gold standard, donor material is difficult to collect and in short supply. Furthermore, patients who receive donated islets require immunosuppression for the rest of their lives, which can significantly impact lifestyle (Latres et al., 2019). The generation of human pluripotent stem cell (hPSC)‐derived islets (SC‐islets) could provide a renewable and unlimited source of β‐cells that no longer require donors and have the potential to bypass immunosuppression if the stem cells came from the same patient.

Recent reports highlight the potential of stem‐cell therapies in combating the complications and struggles that many patients with T1D encounter in their everyday lives (Wang et al., 2024). In the past several years, many groups have focused on optimizing SC‐islet protocols to improve the scalability, reproducibility, and functionality of insulin‐secreting β‐cells (Balboa et al., 2022; Barsby et al., 2022; Jarc et al., 2024; Mahaddalkar et al., 2020; Pagliuca et al., 2014; Pollock et al., 2023; Rezania et al., 2014; Sali et al., 2025; Zanfrini et al., 2024). Importantly, this research has also demonstrated that between 2 × 10^6^ and 5 × 10^6^ SC‐islet cells suffice to reverse diabetes in mice, whereas 750,000 SC‐islet cells are insufficient (Maxwell et al., 2022). This work indicates that roughly 10^9^ SC‐islet cells might be needed to treat a person living with T1D. Many groups in both academia and industry are currently focusing on the scalability of this treatment option because of the excitement surrounding SC‐islets as an alternative to islet transplantation. Protocols such as those presented here could alleviate the need for more donor islets and could even be used with patient‐derived stem cells to generate autologous SC‐islets, further improving therapeutic treatment for people living with T1D (Reichman et al., 2025; Swerdlow et al., 1985).

The SC‐islet differentiation protocols we present here have been optimized to include two‐ and three‐dimensional culture methods, characterization methods to validate all differentiation stages, and detailed examples of proper techniques that might otherwise be overlooked. We also discuss how our protocol has been influenced and inspired by other published protocols (Balboa et al., 2022; Barsby et al., 2022; Braam et al., 2023; Pollock et al., 2023; Sali et al., 2025). While many protocols have unique culturing conditions and rely on different signals, our goal here is to clearly present our reproducible, stepwise protocol for engineering SC‐islets that can be used by both highly trained and junior scientists to create the same insulin producing endocrine cells for studies of islet development and function, with the ultimate goal of improving SC‐islet therapies for treating T1D.

### Critical Parameters

#### Starting with good‐quality hPSCs is essential

Use healthy, compact colonies of hPSCs with minimal to no spontaneous differentiation and confirmed expression of pluripotency markers (OCT3/4 and NANOG).

#### Maintain appropriate cell density

hPSCs should be 70–80% confluent before being seeded for differentiation. Over‐confluent or poorly maintained cultures can compromise the efficiency of differentiation.

#### Control cell dissociation and viability

During differentiation, generate a high‐quality single‐cell suspension with >90% viability. Avoid excessive pipetting or over‐digestion.

#### Follow stage‐specific timing and culture conditions

Use of appropriate culture conditions such as incubation time and temperature during the progression through S1‐S7 is critical for efficient pancreatic lineage specification and SC‐islet formation.

#### Perform quality control checkpoints

Confirm the identity of pancreatic progenitors at the end S4 before proceeding to aggregation.

#### Handle AggreWell clusters carefully

Proper removal of bubbles, correct seeding density, and gentle medium changes are required to generate uniform‐sized clusters with consistent differentiation and minimum cluster loss.

### Troubleshooting

Table 12 lists problems that may arise with this procedure along with their possible causes and solutions.

**Table 12:** Important Troubleshooting Steps in hPSCs to SC‐Islet Differentiation Procedure

Problems	Potential Causes	Troubleshooting Strategies
Poor hPSC survival/attachment after thawing	Cell stress during thawing or over pipetting	Minimize handling and maintain cells in colonies with Y‐27632 during recovery
Increased spontaneous differentiation	Poor hPSC maintenance or over‐confluency	Use healthy colonies and maintain appropriate confluency before differentiation
Low differentiation efficiency	Poor starting hPSC quality or incorrect stage conditions such as incubation time and temperature	Confirm pluripotency at the start and follow stage‐specific medium preparation and timing
Low cell viability after dissociation	Excessive enzymatic digestion or mechanical stress (handling)	Optimize dissociation conditions and minimize pipetting
Poor PP formation	Inefficient S4 differentiation	Confirm PP marker expression before aggregation
Uneven or abnormal cluster formation	AggreWell bubbles or improper cell mixing	Remove bubbles and carefully mix cells during seeding
Loss of clusters during 3D culture	Harsh cluster handing and inadequate pipetting	Aspirate and dispense medium carefully to perform slow and gentle medium changes

### Understanding Results

This protocol provides detailed methods for the thawing, recovery, maintenance, passaging and directed differentiation of hPSCs into SC‐islets under specific in vitro conditions. When thawed and plated using this protocol, hPSCs attach within 24 hr (Fig. 2A, day 1), form compact colonies with smooth borders, and exhibit minimal/no spontaneous differentiation (Fig. 2A, day 4) if maintained properly. Maintained hPSCs retain high viability and proliferative capacity over multiple passages. After passaging, hPSCs display stable and homogeneous expression of the pluripotency markers OCT3/4 and NANOG (Fig. 2C and D).

Upon successful initiation of differentiation, hPSCs efficiently differentiate through early developmental specification stages, including definitive endoderm (DE; S1), primitive gut tube (PGT; S2), posterior foregut (PF; S3), and pancreatic progenitor (PP; S4), as evidenced by high expression of key PP markers such as SOX9, PDX1, and NKX6.1 (Fig. 5). At the end of the PP stage, cells adopt a characteristic epithelial morphology and organize into dense, three‐dimensional structures. Marker expression is generally consistent across differentiations, indicating reproducible patterning toward the pancreatic lineage.

**Figure 7:**
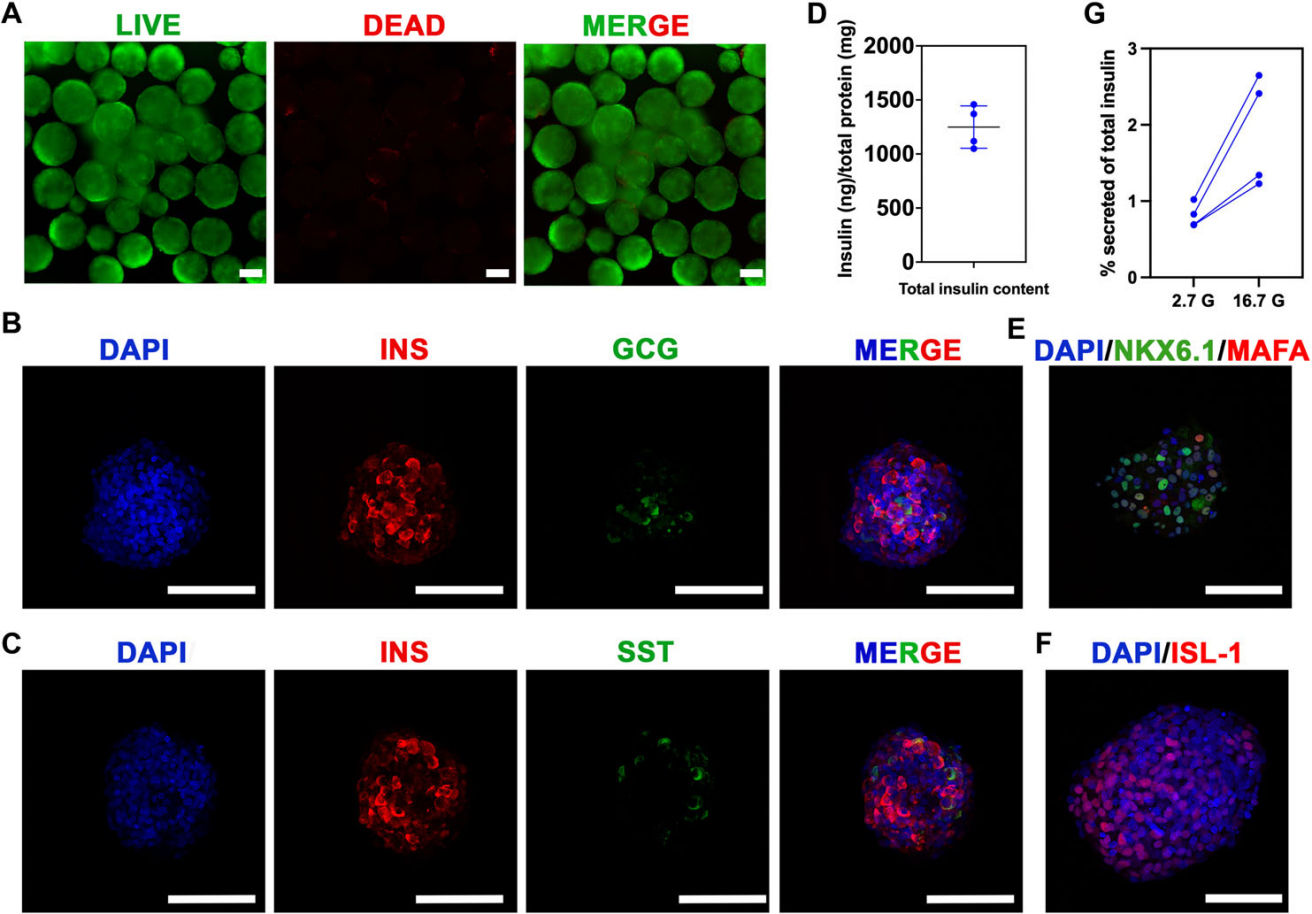
Viability and endocrine maturity of SC‐islets. (**A**) FDA/PI staining showing high cellular viability with SC‐islets. (**B and C**) Single‐channel and merged immunofluorescence (IF) staining of SC‐islets reveals robust expression of key endocrine markers including insulin, glucagon and somatostatin. (**D**) Total insulin content within SC‐islets, normalized to total protein. (**E and F**) Merged IF staining of SC‐islets showing robust expression of maturity markers (MAFA and ISL‐1). (**G**) Insulin secretion response of SC‐islets to increased glucose concentrations: 2.7 mM (2.7 G) and 16.7 mM (16.7 G). IF images are representative merged confocal *z*‐stacks of whole‐mount‐stained SC‐islets; scale bars, 100 µm.

Further differentiation of PPs upon aggregation results in the formation of SC‐islets composed of endocrine cells, including insulin‐positive β‐like cells, glucagon‐positive α‐like cells, and somatostatin‐positive δ‐like cells. SC‐islets exhibit high viability and spheroidal architecture, with endocrine markers spread throughout the cluster (Fig. 7A‐C). Additionally, SC‐islets possess higher intracellular insulin content (Fig. 7D) consistent with native β‐cells. SC‐islets also display partial expression of key maturity markers such as MAFA and ISL‐1 (Fig. 7E and F) and demonstrate robust, glucose‐responsive insulin secretion upon exposure to increased glucose concentrations (Fig. 7G). The SC‐islets generated from these protocols are suitable for numerous downstream applications, including functional assays, imaging‐based analyses, and disease‐modeling studies. For the differentiation process we describe here, we begin with ∼2.5 million hPSCs per well in a 6‐well dish for stages 1‐4. To generate clusters, we add 1.8 million PP cells per well of 24‐well AggreWell dish to generate ∼1,200 clusters/well. A full plate would therefore generate ∼28,800 clusters, each consisting of between 1000 and 1500 cells. These calculations are based on full plates, although it is common practice to use only a subset of wells in each AggreWell dish for a differentiation. If the differentiation is successful and free of contamination, the unused empty wells can also be reused in later differentiations. Though these cell counts and conditions have been optimized specifically for pancreatic endocrine differentiation, the outlined framework may be broadly applicable to other directed differentiation systems.

### Time Considerations

The generation of SC‐islets from hPSCs takes ∼43 days, with the final duration depending on the hPSC line, differentiation efficiency, and experimental goals. Before differentiation, hPSCs typically require two passages (7‐8 days) to recover after thawing. After recovery, cells require an additional 3‐4 days of expansion after passaging to achieve the appropriate cell number for differentiation. Preparation of culture materials such as Matrigel coating and fresh medium requires ∼30 min, depending on the specific preparation steps.

The differentiation process consists of seven stages. Monolayer differentiation from Stage 1 to Stage 4 takes ∼12 days, including preparation for aggregation. 3D culture from Stage 5 to Stage 7 requires 24 days to generate functional SC‐islets.

Following completion of differentiation, additional time and steps may be required for downstream analysis, including quality control, immunostaining, flow cytometry, gene expression analysis, and functional assays such as glucose‐stimulated insulin secretion (GSIS) assays. Therefore, the entire process, from hPSC preparation to mature SC‐islets suitable for functional characterization, typically requires ∼6‐7 weeks, depending on the experimental endpoint and assay requirements.

### Author Contributions

**Manuj Bandral**: Conceptualization; investigation; writing—original draft; methodology; validation; visualization; writing—review and editing; formal analysis; data curation; funding acquisition. **Jimena Alvarez‐Castanon**: Writing—original draft; writing—review and editing. **David S. Lorberbaum**: Conceptualization; funding acquisition; writing—original draft; methodology; visualization; writing—review and editing; formal analysis; supervision; resources.

### Conflict of Interest

The authors declare no conflict of interest.

## Data Availability

The data, tools, and material (or their source) that support the protocol are available from the corresponding author upon reasonable request.
